# Prevalence and correlates of depression and substance use disorders in emergency department populations: A cross-sectional study at East Africa's largest public hospital

**DOI:** 10.1016/j.afjem.2022.06.008

**Published:** 2022-07-20

**Authors:** Theddeus Iheanacho, Kaitlin R. Maciejewski, Frances Ogudebe, Faith Chumo, Tracie Slade, Rebecca Leff, Christine Ngaruiya

**Affiliations:** a Department of Psychiatry, Yale University School of Medicine, New Haven, CT, USA; b Yale Center for Analytical Sciences, Yale University School of Public Health, New Haven, CT, USA; c Yale University School of Nursing, New Haven, CT, USA; d Department of History of Science, Medicine and Public Health, Yale College, New Haven, CT, USA; e Dornsife School of Public Health, Drexel University, Philadelphia, PA, USA; f Department of Emergency Medicine, Mayo Clinic, Rochester, MN, USA; g Department of Emergency Medicine, Yale University School of Medicine, New Haven, CT, USA

**Keywords:** Mental health, Depression, Substance use disorder, Alcohol, Tobacco, Emergency department

## Abstract

**Introduction:**

There are persistent gaps in screening, identification, and access to care for common mental disorders in Low- and Middle-Income Countries. An initial step towards reducing this gap is identifying the prevalence, co-morbidities, and context of these disorders in different clinical settings and exploring opportunities for intervention. This study evaluates the prevalence and correlates of depression and substance use disorders among adults presenting to the Emergency Department (ED) of a major national hospital in East Africa.

**Methods:**

This study utilized the World Health Organization's STEPwise Approach to Surveillance (WHO-STEPS) tool and the Patient Health Questionnaire (PHQ-9) to conduct a cross-sectional survey capturing socio-demographic data, tobacco, and alcohol use and rates of depression in a sample of adults presenting to the ED. Bivariate and multivariate analyses were conducted for each outcome of interest and socio-demographics.

**Results:**

Of 734 respondents, 298 (40.6%) had a PHQ-9 score in the “moderate” to “severe” range indicative of major depressive disorder. About 17% of respondents endorsed current tobacco use while about 30% reported being daily alcohol users. Those with high PHQ-9 score had higher odds of reporting current tobacco use (“severe range” = adjusted odds ratio (aOR) 1.85, 95% CI 1.05, 3.26). Those with a “severe” PHQ-9 scores were 9 times (aOR 2.3-35.3) more likely to be daily drinkers.

**Conclusions:**

Screening and identification of people with depression and substance use disorders in the ED of a large national hospital in Kenya is feasible. This offers an opportunity for brief intervention and referral to further treatment.

## African relevance

• This study adds to the body of work identifying the burden of mental disorders in Low- and middleincome countries (LMICs) in Africa.
• It explores opportunities and strategies for increasing access to mental health care reducing morbidityand mortality.
• Our findings could guide government health agencies, policy makers, research organisations and local foundations as they work together to tackle the growing public mental health need in Kenya and potentially in other LMICs especially in sub-Saharan Africa.

## Introduction

Non-Communicable Diseases (NCDs), defined by the World Health Organization (WHO) as “chronic diseases that are not passed from person to person” including mental and substance use disorders [1], are the leading cause of death globally accounting for 70% of the world's 56 million deaths [1]. Nearly half of deaths due to NCDs occur prematurely [2]. This burden of disease continues to rise in Low- and Middle income countries (LMICs) [3,4].

In Sub-Saharan Africa, 34% of deaths were due to NCDs in 2015 [5]. Constituting a similar worrisome burden, in Kenya, NCDs account for more than 50% of total hospital admissions and over 55% of hospital deaths [6]. The Kenya STEPS report demonstrated that the continued rise in burden of NCDs has the potential to produce devastating economic effects [7].

Mental disorders, including substance use disorders, are one of the leading causes of morbidity among all NCDs [8]. Mental disorders are associated with increase in mortality and poorer health outcomes, especially in LMICs [8]. High rate of unhealthy substance use, particularly alcohol use, has been well documented among adults presenting with injuries in African countries like Ghana [9,10], Malawi [11] and Mozambique[12] and South Africa[13]. The interface between mental illness, substance use disorders and other NCDs, is well-established in studies from High-Income Country settings [14], [15], [16]. While these initial studies provide key insights into the bidirectional relationship between these disorders and other NCDs, more data is needed from LMICs to address the persistent burden of diseases due to mental and substance use disorders and treatment gaps that exist [8,[14], [15], [16], [17], [18]].

There is still a dearth in resources allocated towards combating mental and substance use disorder in most LMICs [19], [20], [21], [22]. Clearer government policies and interventions specifically addressing risk factors for mental disorders in the context of other NCDs have been shown to help achieve early identification, treatment, and better outcomes [23,24]. Such policies and interventions are urgently needed in LMICs particularly in Sub-Saharan Africa. Decreasing the burden of disease due to mental illness, substance use and other NCDs is in line with the World Health Organization's Sustainable Development Goals and must be prioritized by governments and health agencies [25], [26], [27]. An initial step towards this goal is identifying the prevalence, co-morbidities, and context of these disorders in different clinical practice settings and exploring opportunities for intervention.

This study explores the prevalence and correlates of depression and substance use disorders among adults presenting to the Emergency Department of a major national hospital in East Africa.

## Methods

### Study design

This was a cross-sectional, observational study [28] that used the World Health Organization's STEPwise Approach to Surveillance (WHO-STEPS) tool to obtain socio -demographic data as well as, tobacco and alcohol use and the Personal Health Questionnaire (PHQ-9) to assess prevalence of depression in a sample of adults presenting to the emergency department for acute care [29,30]. The WHO-STEPS tool and PHQ-9 are both validated and widely used tools, with proven relevance for LMICs [31], [32], [33], [34]. Two data collectors trained in qualitative methods and interviewing were hired and received a two-week onboarding regarding the study protocol and study recruitment site. This was done by the principal investigator, with quality assurance by an onsite research coordinator familiar with the clinical site. The data collectors were familiar with similar patient populations and spoke both English and the native languages fluently and thus were able to translate the questions when needed. The survey was verbally administered to account for potential barriers with illiteracy, with responses indicated on electronic tablets. Survey responses were recorded on the Kobo™ software [35] platform given usability even when internet is limited. The surveys were offered in English and in the national language, Kiswahili. This study received approval from the Institutional Review Board at Yale University (IRB Protocol ID 2000022697) and from the Kenyatta National Hospital/ University of Nairobi Ethics Review Committee (study registration No. A&E/034/201).

### Setting

The Kenyatta National Hospital is a major tertiary care center in Kenya and the largest hospital in East Africa. An estimated 14,956 to 23,951 patients were seen at the Kenyatta National Hospital Emergency Department (KNH ED) in a three month period between 2014 and 2015, according to Myers et al. [36].

### Sample

We had a target sample size of 2400 or 10% of the upper estimate of total number of presentations based on the hospital's historical three month patient visit data referenced above, in line with standard pilot proportions [37]. However, because of unforeseen system changes during the period of our study, a large proportion of patients accessing care at the KNH ED were referred away significantly reducing the patient volume. In lieu of our original study plan, we then extended data collection to the entirety of the funding period, for a total of six months and included all patients meeting our inclusion criteria. All patients aged 18 years old or older were eligible for the study. All adults in this age group presenting to the ED within the period of the study were screened and offered participation in the study. This mirrored the age group of participants in the 2015 Kenya WHO National STEPwise approach to Surveillance (STEPs) study co-led by co-author (CN) and others [7,33,34]. Patients who could not provide informed consent for any reason including cognitive impairment, severe injury, or acute intoxication, were excluded.

### Measures

The WHO-STEPS, included items on self-reported sociodemographic information including age in years, sex and marital status. Participants were also asked about their highest education level. To collect employment information, participants were asked “which of the following best describes your main work status over the past 12 months?” Responses were then categorized as: employed (government employee, non-government employee), not employed (homemaker, retired, non-paid, unemployed, and able to work, unemployed and unable to work), student and self-employed. To gain information about income, participants were asked “Taking the past year, can you tell me what the average earnings of the household have been?” They were also asked “How many people older than 18 years, including yourself, live in your household?”. These variables were then used to create an indicator for household poverty, using the WHO standard [38]. The household was in poverty if household income divided by household size was less than 69397.5 KES (given a poverty cut-off of $1.90 per person per day, $1.9 * 365.25days * 100KES/$ = 69397.5 KES). The survey items on tobacco and alcohol use captured data on current use, rates of use, historical use, and impact on functioning **(Supplemental material 1).** The Patient Health Questionnaire (PHQ-9) is a nine-item multipurpose instrument for screening, diagnosing, monitoring, and measuring the severity of depression. It incorporates the Diagnostic and Statistical Manual of Mental Disorders (DSM-V) criteria for major depressive disorder into a brief self-report tool. The total score for the nine items ranges from 0 to 27. Scores of 5, 10, 15, and 20 represent cutoff points for mild, moderate, moderately severe and severe depression, respectively [39,40]. A score of 10 or greater represents current depression **(Supplemental material 2).**

### Statistical analysis

Descriptive statistics were used to characterize the study population. Mean and standard deviation were calculated and presented for numerical variables, number and percentages were presented for categorical variables, and number missing were presented for all.

Bivariate analyses were conducted for each outcome of interest and demographics. Numeric variables were compared using t-test or ANOVA and chi-squared test or fisher's exact test were used to compare differences between categorical variables, as appropriate. Outcomes included PHQ-9 score category; tobacco use including lifetime use, current use, current smoker, current smokeless tobacco user, daily smoker, or daily smokeless tobacco user; alcohol use including lifetime use, daily drinker, frequency of being unable to stop drinking, frequency of when drinking has affected responsibilities, frequency of needing eye-opener, frequency of drinking causing trouble with relationships, ever stopped drinking due to health reasons. Each question of the PHQ-9 scale was included as a binary outcome (yes vs no). Demographic comparisons included participant age in years, household income in past year (KES), number of adults (> 18 years) living in household, participant sex, education level, marital status, type of work, current depression as indicated by a score of at least 10 on the PHQ-9, and indicator of household poverty.

Multivariable analyses were conducted using covariate-adjusted logistic analysis. Association of socioeconomic status with mental illness was conducted by using PHQ-9 score category as outcome. The reference category was no depression and outcomes included mild, moderate, moderately severe, and severe depression. Models were adjusted for age in years, gender (female vs male), education (less than primary school vs completed secondary or above; completed primary school vs completed secondary or above), marital status (formerly married vs never married; married vs never married), work status (employed vs unemployed; self-employed vs unemployed; student vs unemployed), and household poverty (yes vs no). Association of depression with substance use included the bivariate outcomes lifetime use of tobacco (yes vs no), current tobacco user (yes vs no), lifetime alcohol use (yes vs no), daily alcohol drinker (yes vs no). All models were adjusted for age in years, and PHQ-category mild, moderate, moderately severe, severe, with none as the reference. Additional models for association of depression and substance use with NCD's included the bivariate outcomes of ever been told they had hypertension (yes vs no), ever been told they had raised blood sugar or diabetes (yes vs no), history of cardiovascular disease (yes vs no), history of hyperlipidemia (yes vs no). These models were adjusted for PHQ-9 category mild, moderate, moderately severe, severe, with none as the reference, lifetime tobacco use (yes vs no), and lifetime alcohol use (yes vs no). Association of depression and substance use with likelihood of NCD management was investigated by limiting the sample to those who answered “yes” to the appropriate disease for the following outcomes of have taken medication for hypertension (yes vs no), diabetes (yes vs no), taking aspirin (yes vs no), or statin (yes vs no). These models were adjusted for PHQ-9 category and tobacco and alcohol use as in the previous models.

A p-value less than 0.05 was considered statistically significant. Analyses were conducted using SAS 9.4 (SAS Institute, Inc., Cary, NC).

### Role of the funding source

The funders of the study had no role in study design, data collection, data analysis, data interpretation, or writing of the report.

## Results

There was a total of 923 total eligible respondents, of whom 734 (79.5%) provided consent and completed the survey questions, including the PHQ-9 items**.** The mean age was 35 years (*sd 13)*. Most respondents were male (60.9%, *n* = 447/734). There were no significant differences on these characteristics between those who consented and completed the survey and those who did not. A vast majority of respondents had completed at least primary school (86.7%, *n* = 636/734). More than half reported being married (54.6%, *n* = 401/734), and a third of respondents had never been married (36.2%, *n* = 266/734). Majority reported being self-employed (41.3%, *n* = 303/734), and one in five reported being unemployed (20.2%, *n* = 148/734). The average reported annual household income was Kenya Shillings (KES) 238,241.82 (sd 271,536.17), approximately 2,382.41 (sd 2,715.40) USD.

Overall, 40.6% (*n* = 298/734) of respondents had a PHQ-9 score in the “moderate” to “severe” range, indicating the need for an intervention for depression. Specifically, 10.1% (*n* = 74/734) of respondents were in the “severe” range. The mean age of those with a score of “severe” was 40 years (*sd 11*). The predominant sex reporting a “severe” score was male, 56.8% (*n* = 42/74), reflective of the demographic distribution of the sample population. Those with a primary school education level constituted the highest frequency of those with a “severe” score, 39.2% (*n* = 29/74). Furthermore, the majority of those with a “severe” score were married (50%, *n* = 37/74), self-employed (46.6%, *n* = 34/73), and were below the poverty line (61.1%, *n* = 22/35) (Table 1).

**Table 1:** PHQ-9 score by sociodemographics among participants at KNH ED.

Mean (SD) or N (%)	PHQ-9 score				
	None (*N* = 202)	Mild (*N* = 234)	Moderate (*N* = 144)	Moderately Severe (*N* = 80)	Severe (*N* = 74)
**Age (yr)**	32.48 (13.06)	33.02 (11.59)	37.21 (13.32)	39.65 (14.66)	39.86 (11.34)
**Household income in past year (kSh)**	231625 (231873.3)	202500.23 (214324.64)	198380 (168726.67)	155277.78 (188833.35)	307815.6 (368163.52)
**Number adults (18+) living in household**	2.60 (1.32)	2.74 (2.52)	2.60 (1.67)	2.80 (2.29)	2.38 (1.41)
**Sex**					
Female	76 (37.81%)	74 (31.62%)	66 (45.83%)	37 (46.84%)	32 (43.24%)
Male	125 (62.19%)	160 (68.38%)	78 (54.17%)	42 (53.16%)	42 (56.76%)
**Education**					
Less than primary school	12 (5.94%)	22 (9.48%)	26 (18.06%)	18 (22.50%)	18 (24.32%)
Primary school completed	44 (21.78%)	54 (23.28%)	45 (31.25%)	23 (28.75%)	29 (39.19%)
Secondary school or above	146 (72.28%)	156 (67.24%)	73 (50.69%)	39 (48.75%)	27 (36.49%)
**Marital Status**					
Formerly Married	6 (2.99%)	10 (4.31%)	18 (12.59%)	13 (16.25%)	16 (21.62%)
Married	111 (55.22%)	128 (55.17%)	83 (58.04%)	42 (52.50%)	37 (50.00%)
Never married	84 (41.79%)	94 (40.52%)	42 (29.37%)	25 (31.25%)	21 (28.38%)
**Type of work**					
Employed	55 (27.23%)	70 (30.17%)	36 (25.17%)	16 (20.00%)	13 (17.81%)
Self-employed	71 (35.15%)	97 (41.81%)	62 (43.36%)	39 (48.75%)	34 (46.58%)
Student	39 (19.31%)	33 (14.22%)	8 (5.59%)	6 (7.50%)	3 (4.11%)
Unemployed	37 (18.32%)	32 (13.79%)	37 (25.87%)	19 (23.75%)	23 (31.51%)
**Household poverty?**					
No	91 (64.54%)	86 (56.95%)	47 (43.93%)	23 (46.00%)	14 (38.89%)
Yes	50 (35.46%)	65 (43.05%)	60 (56.07%)	27 (54.00%)	22 (61.11%)

Regarding substance use, 37.3% (272/730) of respondents reported having ever used tobacco (smoked or smokeless), and 16.8% (*n* = 104/618) reported being current tobacco users. Those reporting current tobacco use had an average age of 37.6 yr (*sd 12.5*), were predominantly male (92.3%, *n* = 96/104), had a primary school education or less (58.2%, *n* = 60/104), and were married (47.6%, *n* = 49/104) **(**Table 2).

**Table 2:** Patterns of tobacco use in KNH ED patients.

Mean (SD) or N (%)	Ever used tobacco		Current tobacco user		Current tobacco smoker		Current smokeless tobacco user			Daily smoker	
	No (*N* = 458)	Yes (*N* = 272)	No (*N* = 514)	Yes (*N* = 104)	No (*N* = 643)	Yes (*N* = 91)	No (*N* = 527)	Yes (*N* = 15)	P-value	No (*N* = 19)	Yes (*N* = 71)
**Age (yr)**	33.12 (12.06)	38.37 (13.87)	33.74 (12.74)	37.36 (12.50)	34.61 (13.00)	38.50 (12.43)	33.89 (12.77)	29.43 (9.85)	0.2	35.32 (12.72)	39.47 (12.35)
**Household income in past year (kSh)**	241481.48 (282647.25)	228613.65 (247517.76)	229665.66 (272878.35)	224571.79 (249303.72)	239305.42 (273550.18)	230968.15 (259357.48)	228014.79 (271223.31)	155555.56 (154229.63)	0.43	205692.31 (106698.47)	237673.98 (287087.42)
**Number adults (18+) living in household**	2.68 (1.60)	2.59 (2.44)	2.73 (1.73)	2.45 (3.16)	2.68 (1.69)	2.40 (3.25)	2.72 (1.72)	3.07 (2.71)	0.63	3.16 (6.54)	2.20 (1.50)
**Sex**									0.09		
Female	239 (52.41%)	44 (16.18%)	257 (50.20%)	8 (7.69%)	280 (43.68%)	5 (5.49%)	257 (48.95%)	4 (26.67%)		0 (0.00%)	5 (7.04%)
Male	217 (47.59%)	228 (83.82%)	255 (49.80%)	96 (92.31%)	361 (56.32%)	86 (94.51%)	268 (51.05%)	11 (73.33%)		19 (100.00%)	66 (92.96%)
**Education**									0.12		
Less than primary school	37 (8.10%)	59 (21.77%)	47 (9.16%)	31 (30.10%)	68 (10.59%)	28 (31.11%)	50 (9.51%)	4 (26.67%)		1 (5.26%)	27 (38.57%)
Primary school completed	116 (25.38%)	78 (28.78%)	134 (26.12%)	29 (28.16%)	168 (26.17%)	27 (30.00%)	137 (26.05%)	3 (20.00%)		5 (26.32%)	21 (30.00%)
Secondary school or above	304 (66.52%)	134 (49.45%)	332 (64.72%)	43 (41.75%)	406 (63.24%)	35 (38.89%)	339 (64.45%)	8 (53.33%)		13 (68.42%)	22 (31.43%)
**Marital Status**									0.2		
Formerly Married	29 (6.37%)	34 (12.55%)	34 (6.65%)	17 (16.50%)	48 (7.50%)	15 (16.67%)	34 (6.49%)	2 (13.33%)		2 (10.53%)	13 (18.57%)
Married	250 (54.95%)	147 (54.24%)	280 (54.79%)	49 (47.57%)	356 (55.63%)	45 (50.00%)	290 (55.34%)	5 (33.33%)		10 (52.63%)	34 (48.57%)
Never married	176 (38.68%)	90 (33.21%)	197 (38.55%)	37 (35.92%)	236 (36.88%)	30 (33.33%)	200 (38.17%)	8 (53.33%)		7 (36.84%)	23 (32.86%)
**Type of work**									0.58		
Employed	118 (25.88%)	70 (25.93%)	130 (25.44%)	25 (24.27%)	169 (26.41%)	21 (23.33%)	135 (25.76%)	4 (26.67%)		4 (21.05%)	17 (24.29%)
Self-employed	189 (41.45%)	112 (41.48%)	209 (40.90%)	46 (44.66%)	261 (40.78%)	42 (46.67%)	214 (40.84%)	4 (26.67%)		9 (47.37%)	32 (45.71%)
Student	74 (16.23%)	15 (5.56%)	80 (15.66%)	4 (3.88%)	88 (13.75%)	1 (1.11%)	80 (15.27%)	3 (20.00%)		1 (5.26%)	0 (0.00%)
Unemployed	75 (16.45%)	73 (27.04%)	92 (18.00%)	28 (27.18%)	122 (19.06%)	26 (28.89%)	95 (18.13%)	4 (26.67%)		5 (26.32%)	21 (30.00%)
**Current Depression (PHQ >= 10)**									0.87		
No	287 (62.66%)	147 (54.04%)	315 (61.28%)	63 (60.58%)	381 (59.25%)	55 (60.44%)	327 (62.05%)	9 (60.00%)		10 (52.63%)	44 (61.97%)
Yes	171 (37.34%)	125 (45.96%)	199 (38.72%)	41 (39.42%)	262 (40.75%)	36 (39.56%)	200 (37.95%)	6 (40.00%)		9 (47.37%)	27 (38.03%)
**Household poverty?**									0.75		
No	154 (52.03%)	104 (56.22%)	168 (50.76%)	43 (61.43%)	222 (52.48%)	39 (62.90%)	170 (50.45%)	4 (44.44%)		9 (69.23%)	30 (61.22%)
Yes	142 (47.97%)	81 (43.78%)	163 (49.24%)	27 (38.57%)	201 (47.52%)	23 (37.10%)	167 (49.55%)	5 (55.56%)		4 (30.77%)	19 (38.78%)

On alcohol use, most of the respondents, 61.2% (446/729) reported having ever used alcohol, and 29.5% (215/729) were daily alcohol drinkers. Those reporting daily alcohol use had average age of 30.9 yr (sd *9.8*), were mostly male (76.3%, *n* = 164/215) and most had higher education (72.7%, *n* = 157/215). Those who reported stopping drinking due to health reasons (33.2%, *n* = 62/187), were most likely to live in a larger household with average of 3.35 people (sd *2.26*), mostly male (83.9%, 50/62) with most having current depression (64.52%, 40/62) **(**Table 3).

**Table 3:** Patterns of alcohol use by sociodemographics among participants in KNH ED patients.

Mean (SD) or N (%)	Ever used alcohol		Daily Drinker		Unable to stop drinking					Stopped drinking due to health reasons		
	No (*N* = 283)	Yes (*N* = 446)	No (*N* = 43)	Yes (*N* = 215)	Never (*N* = 81)	Less than monthly (*N* = 10)	Monthly (*N* = 6)	Weekly (*N* = 7)	Daily or almost daily (*N* = 6)	No (*N* = 125)	Yes (*N* = 62)	P-value
**Age (yr)**	33.63 (13.07)	36.01 (12.82)	38.10 (11.03)	30.91 (9.82)	31.88 (11.86)	27.70 (5.44)	37.00 (15.44)	32.14 (7.49)	42.83 (13.61)	40.13 (14.20)	44.00 (13.13)	0.08
**Household income in past year (kSh)**	227896.97 (292897.94)	245290.96 (260759.44)	152066.67 (150948.02)	269013.07 (281973.15)	258963.64 (280486.02)	255000 (190488.09)	119000 (79031.64)	287333.33 (255567.34)	65000.00 (38729.83)	259080.75 (252897.29)	205223.40 (248781.20)	0.24
**Number adults (18+) living in household**	2.69 (1.68)	2.61 (2.11)	2.21 (1.92)	2.50 (2.33)	2.56 (1.51)	2.00 (1.05)	1.33 (0.52)	1.86 (1.07)	2.67 (2.73)	2.56 (1.55)	3.35 (2.26)	0.014
**Sex**												<0.001
Female	168 (59.36%)	116 (26.01%)	1 (2.33%)	51 (23.72%)	24 (30.00%)	1 (10.00%)	2 (33.33%)	1 (14.29%)	0 (0.00%)	53 (42.40%)	10 (16.13%)	
Male	115 (40.64%)	330 (73.99%)	42 (97.67%)	164 (76.28%)	56 (70.00%)	9 (90.00%)	4 (66.67%)	6 (85.71%)	6 (100.00%)	72 (57.60%)	52 (83.87%)	
**Education**												0.85
Less than primary school	32 (11.31%)	63 (14.13%)	13 (30.23%)	16 (7.41%)	4 (4.94%)	0 (0.00%)	0 (0.00%)	0 (0.00%)	2 (33.33%)	21 (16.94%)	12 (19.35%)	
Primary school completed	93 (32.86%)	101 (22.65%)	12 (27.91%)	43 (19.91%)	22 (27.16%)	2 (20.00%)	2 (33.33%)	2 (28.57%)	1 (16.67%)	32 (25.81%)	14 (22.58%)	
Secondary school or above	158 (55.83%)	282 (63.23%)	18 (41.86%)	157 (72.69%)	55 (67.90%)	8 (80.00%)	4 (66.67%)	5 (71.43%)	3 (50.00%)	71 (57.26%)	36 (58.06%)	
**Marital Status**												0.72
Formerly Married	25 (8.87%)	38 (8.54%)	8 (18.60%)	12 (5.56%)	7 (8.64%)	0 (0.00%)	0 (0.00%)	2 (28.57%)	1 (16.67%)	12 (9.76%)	6 (9.68%)	
Married	149 (52.84%)	250 (56.18%)	22 (51.16%)	99 (45.83%)	41 (50.62%)	4 (40.00%)	2 (33.33%)	2 (28.57%)	3 (50.00%)	83 (67.48%)	45 (72.58%)	
Never married	108 (38.30%)	157 (35.28%)	13 (30.23%)	105 (48.61%)	33 (40.74%)	6 (60.00%)	4 (66.67%)	3 (42.86%)	2 (33.33%)	28 (22.76%)	11 (17.74%)	
**Type of work**												0.96
Employed	66 (23.40%)	123 (27.64%)	6 (13.95%)	61 (28.24%)	22 (27.16%)	3 (30.00%)	2 (33.33%)	1 (14.29%)	2 (33.33%)	38 (30.65%)	18 (29.51%)	
Self-employed	115 (40.78%)	188 (42.25%)	26 (60.47%)	85 (39.35%)	31 (38.27%)	5 (50.00%)	3 (50.00%)	4 (57.14%)	3 (50.00%)	51 (41.13%)	26 (42.62%)	
Student	46 (16.31%)	43 (9.66%)	0 (0.00%)	35 (16.20%)	12 (14.81%)	1 (10.00%)	0 (0.00%)	1 (14.29%)	0 (0.00%)	6 (4.84%)	2 (3.28%)	
Unemployed	55 (19.50%)	91 (20.45%)	11 (25.58%)	35 (16.20%)	16 (19.75%)	1 (10.00%)	1 (16.67%)	1 (14.29%)	1 (16.67%)	29 (23.39%)	15 (24.59%)	
**Current Depression (PHQ >= 10)**												0.011
No	167 (58.80%)	267 (59.73%)	17 (39.53%)	159 (73.61%)	48 (59.26%)	7 (70.00%)	5 (83.33%)	1 (14.29%)	4 (66.67%)	69 (55.20%)	22 (35.48%)	
Yes	117 (41.20%)	180 (40.27%)	26 (60.47%)	57 (26.39%)	33 (40.74%)	3 (30.00%)	1 (16.67%)	6 (85.71%)	2 (33.33%)	56 (44.80%)	40 (64.52%)	
**Household poverty?**												0.048
No	83 (50.61%)	178 (55.97%)	16 (53.33%)	98 (64.05%)	27 (49.09%)	6 (75.00%)	4 (66.67%)	6 (100.00%)	1 (25.00%)	47 (54.02%)	17 (36.17%)	
Yes	81 (49.39%)	140 (44.03%)	14 (46.67%)	55 (35.95%)	28 (50.91%)	2 (25.00%)	2 (33.33%)	0 (0.00%)	3 (75.00%)	40 (45.98%)	30 (63.83%)	

There was a statistically significant association between substance use and PHQ-9 scores. Those with severe PHQ-9 score had higher odds of reporting tobacco use (adjusted odds ratio (aOR) 1.85, 95%CI 1.05, 3.26) compared to those with no current depression, adjusting for age and PHQ-9 score. The same was true for those who had a moderate score (aOR 1.7, 95% CI 1.0–2.6). There was also strong evidence of association between being a daily drinker and having high PHQ-9 score. Specifically, after adjusting for age, those with moderate PHQ-9 scores were 6.3 times more likely to be daily drinkers (aOR 6.3, 95% CI 1.8–21.6) compared to those that met no PHQ-9 criteria. Those with a moderately severe PHQ-9 score were 4.4 times more likely (aOR 4.4, 95% CI 1.0–18.7), and those with a severe score were 9 times (aOR 2.3–35.3) more likely to be daily drinkers, respectively (Table 4).

**Table 4:** Association of PHQ-9 with substance use among participants in KNH ED patients.

	Ever used tobacco	Current tobacco user	Ever used alcohol	Daily Drinker
	aOR (95% CI), p-value	aOR (95% CI), p-value	aOR (95% CI), p-value	aOR (95% CI), p-value
Age (yr)	1.03 (1.018, 1.043), <.0001)	1.022 (1.005, 1.038), 0.0097)	1.016 (1.003, 1.029), 0.0128)	1.051 (1.018, 1.084), 0.0021)
PHQ-9 score -mild vs none	1.475 (0.977, 2.226), 0.0645)	1.761 (0.997, 3.111), 0.0512)	1.952 (1.309, 2.909), 0.001)	2.1 (0.637, 6.92), 0.2229)
PHQ-9 score -moderate vs none	1.651 (1.041, 2.618), 0.033)	1.275 (0.654, 2.486), 0.4763)	1.36 (0.87, 2.126), 0.1778)	6.259 (1.817, 21.561), 0.0037)
PHQ-9 score – moderately severe vs none	1.037 (0.583, 1.846), 0.9008)	1.191 (0.533, 2.663), 0.6696)	1.195 (0.692, 2.062), 0.5232)	4.382 (1.028, 18.673), 0.0458)
PHQ-9 score - severe vs none	1.848 (1.049, 3.256), 0.0335)	1.35 (0.587, 3.108), 0.4799)	1.085 (0.624, 1.888), 0.7716)	9.035 (2.315, 35.264), 0.0015)

*Note:* aOR: adjusted odds ratio; CI: confidence interval

## Discussion

Our results showed that there was a high prevalence (40%) of moderate to severe depression in this population of adults presenting to the ED of this large, national hospital as indicated by their scores on the PHQ-9 survey. Substance use was also common among the respondents with almost 17% being current tobacco users, representing a higher prevalence as compared to the Kenya WHO-STEPs study (13.5%). About 30% of respondents were daily alcohol drinkers, compared to the national average in Kenya (12.6%) [33,34].These are important findings indicating that there is potential for identifying people with depression and substance use disorders in the ED, providing an opportunity for brief intervention or initiating treatment and connecting them to further treatment in the hospital or community. Our findings are also in keeping with results from studies in similar settings in Africa. A study at Tanzania's national hospital ED by Mundega et al showed a high prevalence of substance use. Of the total patient population screened, 46.9% were positive for alcohol, 36.1% were positive for drugs and 26.1% were positive for both alcohol and drugs [41]. Another comparable study in a major South African hospital ED showed a similar trend. Of the total study population enrolled, 37% met criteria for substance use disorder of which 68% were positive for alcohol use disorder alone, 14% for alcohol and drug use and 18% for drug use alone [42].

We also found that substance use was significantly associated with moderate and severe depression as measured by the PHQ-9. It is unclear in this study if substance use led to more depression or if depression led to substance use. However, the relationship between substance use and depression prevalence, treatment, and outcomes have been well documented [43], [44], [45]. The co-existence of both substance use disorder and depression leads to more severe symptoms and worse outcomes for both disorders [43]. At the same time, addressing either or both leads to symptom reduction and better outcomes [44,45]. Therefore, efforts to reduce the burden of NCDs related to substance use and depression should include packages of care that screen for and offer treatment for both.

Emergency care settings represent important entry portals into the healthcare system [46,47]. Given the high prevalence of both depression and substance use amongst adult ED patients identified in our study, the delivery of effective brief interventions for these disorders in the ED has the potential to have a sizeable public health impact [46,47]. Screening, brief intervention, and referral to treatment (SBIRT) is an evidence-based approach to the delivery of early intervention and treatment to people with substance use disorders with demonstrated effectiveness in reducing substance use in emergency department settings in high income countries, with limited data derived from LMICs [42,47,48]. Barriers to implementation cited internationally have included inadequate referral resources, time constraints, poor training, negative provider attitudes, perceptions of role incompatibility, and competing priorities amongst limited healthcare staff [42,49]. These barriers may have relevance in LMICs like Kenya, who face a shortage of mental health professionals. However, training in SBIRT and task-sharing approaches may promote solutions for SBIRT implementation in emergency department settings in LMICs [42,50,51]. Further research is needed to assess the feasibility and effectiveness of SBIRT implementation in acute care settings for mental and substance use disorders in LMICs to address these challenges and promote the adaptation of SBIRT to low resource settings.

Most of the study population completed only primary or secondary/high school education, which is to be expected given that this is a public hospital that provides care to those of lower socioeconomic status in the country. Having a higher education was also associated with knowledge of one's diagnosis. These findings suggest that any intervention for mental disorders designed to be delivered in a public sector tertiary hospital such as the Kenyatta National Hospital should include low-literacy content and psycho-educational materials.

This study was conducted among adults presenting acutely for general medical problems at the ED of large public hospital. Therefore, our findings among this study population may not be generalizable to the general population. However, our study provides a snapshot into the burden of mental disorders in a high-risk and under-addressed population that seeks care in the ED. Additionally, the study population may have higher prevalence of comorbidities. Yet, in selecting a pilot study site, we thought that this site was likely to provide one of the most optimal sites to engage patients presenting from across the region, and our results indicate high disease prevalence in a major public hospital ED with a large population and catchment area. There was also a funding limitation. This study was supported by a pilot fund that limited our ability to extend recruitment and data collection to one year to increase the sample size. However, we were able to provide a snapshot of the burden of depression and substance use disorders in this large hospital ED.

## Conclusions

There is a high prevalence of depression among adults presenting to the Emergency Department (ED) of this large, national hospital. Alcohol use and cigarette smoking were also higher than the national average. These findings indicate that there is potential for identifying people with depression and substance use disorders in the ED, providing an opportunity for brief intervention and connecting them to further treatment in the hospital or community.

## Dissemination plan

Results of the study will be presented to different stakeholders including clinical teams at the national hospital in Kenya where it was conducted during their clinical grand rounds. We will also present at emergency medicine conferences in Africa and internationally.

## Authors’ contributions

Authors contributed as follow to the conception or design of the work; the acquisition, analysis, or interpretation of data for the work; and drafting the work or revising it critically for important intellectual content: TI and CN contributed 25% each and FC, TS, RL, FO and KM contributed 10% each. All authors approved the version to be published and agreed to be accountable for all aspects of the work.

## Declaration of Competing Interest

The authors disclose receipt of the following financial support for the research, authorship, and publication of this article: This work was supported by the Hecht-Albert Global Health Pilot Innovation Award for Junior Faculty, Global Health Leadership Institute, Yale University awarded to CN. The funders had no role in study design, data collection and analysis, decision to publish, or preparation of the manuscript. Funder website: https://medicine.yale.edu/news-article/14993/. The authors declared no other conflicts of interest.
